# Pretreatment neutrophil‐to‐lymphocyte ratio as a potential prognostic biomarker for newly diagnosed patients with metastatic castration‐sensitive prostate cancer

**DOI:** 10.1002/cnr2.1392

**Published:** 2021-06-22

**Authors:** Samer Salah, Ramiz Abu‐Hijlih, Fawzi Abuhijla, Faris Tamimi, Abdallah Al‐Tell, Mohammed Shahait

**Affiliations:** ^1^ Department of Medical Oncology King Hussein Cancer Center Amman Jordan; ^2^ Department of Radiation Oncology King Hussein Cancer Center Amman Jordan; ^3^ Department of Surgery, Division of Urology King Hussein Cancer Center Amman Jordan

**Keywords:** biomarkers, metastasis, prognosis, prostate cancer, survival

## Abstract

**Background:**

Although the prognostic role of neutrophil‐to‐lymphocyte ratio (NLR) has been assessed in patients with metastatic castration‐resistant prostate cancer, data on its impact on oncological outcomes of patients with metastatic castration‐sensitive prostate cancer (mCSPC) are scarce.

**Aim:**

This study aims to examine the influence of elevated pretreatment NLR on time to prostatic‐specific antigen (PSA) progression and overall survival (OS) of patients with mCSPC.

**Methods:**

We retrospectively reviewed patients presenting between June 2007 and June 2019 with mCSPC. Survival was estimated by the Kaplan‐Meier method and compared by the log‐rank test. Multivariate analyses were used to assess the factors influencing time to PSA progression and OS.

**Results:**

A total of 189 patients were included; median age = 69 years, median PSA = 155 ng/mL, 41(22%) had visceral metastasis. Median time to PSA progression was shorter for patients with NLR ≥4 (*n* = 37) compared to patients with NLR < 4 (*n* = 146); 11.3 and 18.3 months, respectively, *P* = .015. Patients with NLR ≥4 also had inferior median OS (23.9 vs 49.5 months, *P* = .001). On multivariate analysis, NLR ≥4 was not an independent factor for time to PSA progression. However, NLR ≥4 was an independent factor of inferior OS (HR: 2.75, 95% CI: 1.01‐7.87, *P* = .047). Other independent factors predicting inferior OS included Eastern Cooperative Oncology Group Performance Status ≥1, high‐volume status, and Hb < 12.

**Conclusion:**

Elevated pretreatment NLR independently predicts inferior OS in newly diagnosed patients with mCSPC. However, NLR was not a predictor of time to PSA progression.

## 1 INTRODUCTION

Prostate cancer is the most common malignancy and the second cause of cancer‐related death among men.^1^, ^2^ According to the American cancer society estimations, 191 930 new cases will be diagnosed and 33 330 will die of the disease in 2020.^1^ Although 5% of prostate cancer patients present with stage IV disease in the USA, one‐fifth of men with prostate cancer present with metastatic disease in the Middle East.^2^

The treatment algorithm of newly diagnosed metastatic castrate‐sensitive prostate cancer (mCSPC) has changed.^3^, ^4^, ^5^, ^6^ Early introduction of novel therapies such as docetaxel, abiraterone, and enzalutamide in the treatment regimen for mCSPC has resulted in significant improvement in overall survival (OS), particularly for the groups with poor prognostic factors.^7^, ^8^ The survival of the CHAARTED‐trial defined high‐volume group is significantly improved with upfront docetaxel. No such benefit was observed for patients with low volume disease.^7^ Similar survival benefit has been reported with upfront use of abiraterone and prednisone plus androgen deprivation therapy (ADT) compared to ADT alone for the LATITUDE‐trial defined high‐risk group.^8^ Several poor prognostic factors in mCSPC have been described such as disease volume, visceral metastasis, Gleason score (GS) ≥8, high serum alkaline phosphatase (ALP), and poor Eastern Cooperative Oncology Group performance status (ECOG PS).^9^, ^10^, ^11^, ^12^, ^13^

Inflammation is a hallmark of cancer development and progression.^14^ Preclinical studies have pointed out that inflammation is involved in various cancer development stages, including initiation, promotion, malignant conversion, invasion, and metastasis.^15^ The neutrophil‐to‐lymphocyte ratio (NLR) is an inflammatory parameter that has been evaluated as a potential prognostic factor in various malignancies, including prostate cancer.^16^ Several reports showed an association between high NLR and poor survival outcomes for many cancers.^16^, ^17^, ^18^ Yin et al conducted a meta‐analysis and found that elevated NLR predicted poor OS (HR  =  1.57; 95% CI 1.41‐1.74; *P*  <  .001) and progression‐free survival (HR  =  1.97; 95% CI 1.28‐3.04; *P*  =  .002) of patients with metastatic castration‐resistant prostate cancer (mCRPC). On the contrary, NLR was not associated with oncological outcomes in patients with localized disease.^19^

Although the impact of NLR on OS has been well studied in metastatic castration‐resistant disease, it is not explored in newly diagnosed patients with mCSPC.^19^, ^20^, ^21^, ^22^, ^23^

The CHAARTED and LATITUDE trials highlighted the importance of prognostic stratification on initial therapy selection.^7^, ^8^ Identifying all prognostic factors at the time of diagnosis of metastatic disease is a pre‐requisite to better risk‐stratify patients enrolled in future trials and may improve therapy selection. In this study, we assessed the prognostic role of pre‐treatment (baseline) NLR in newly diagnosed patients with mCSPC.

## 2 METHODS

### 2.1 Aim and objectives

This study aims to examine the prognostic role of NLR on the oncological outcomes of patients with newly diagnosed mCSPC. Study primary endpoints are to assess the impact of high NLR on time to prostatic‐specific antigen (PSA) progression and OS.

#### 2.1.1 Patients population

After Institutional Review Board approval, we identified all patients with metastatic prostate cancer who were referred to our center between June 2007 and June 2019. All patients were required to have pathologically confirmed prostatic adenocarcinoma and to have a metastatic disease detected on conventional imaging studies (computed tomography (CT) scan, magnetic resonance imaging, and bone scan). Patients with metastasis detected only on prostate‐specific membrane antigen scan without being detected on conventional imaging studies were excluded. Patients who develop a metastatic disease while being treated with ADT for biochemical relapse following definitive primary radiotherapy or radical prostatectomy or those presenting with castration‐resistant metastatic disease were also excluded.

*Variables*: age at presentation, date of diagnosis of metastatic disease, sites of metastasis, number of metastatic bone lesions, GS, ECOG PS, and date of initiation of ADT, defined as the date of the first injection in patients treated with medical castration (GnRH agonists) or the date of orchidectomy for patients treated with surgical castration were abstracted. Concurrent administration of docetaxel and abiraterone was noted. Finally, pre‐treatment laboratory values, including blood counts, serum PSA, ALP, and serum calcium, were extracted. The pretreatment neutrophil‐to‐lymphocyte ratio was calculated for every patient by dividing the absolute neutrophil over the absolute lymphocyte count from the CBC before commencing ADT treatment.

Also, we extracted data on the time of first PSA progression after starting ADT. PSA progression was defined according to the prostate cancer working group criteria (PCWG3).^24^ As such, time to PSA progression was defined as the time from start of ADT to the first PSA increase that is ≥25% and ≥2 ng/mL above the nadir, which is confirmed by a second value ≥3 weeks later. We also gathered data on dates of last follow‐up or death for all patients. OS was defined as time from diagnosis of the metastatic disease until the last follow‐up or death date.

Metastatic burden was stratified into high volume and low volume disease based on the definition from CHAARTED trial. Accordingly, high volume disease was defined as the presence of more than three metastatic bone lesions with at least one lesion outside the spine and pelvis or the presence of visceral metastasis,^7^ while the LATITUDE trial defined risk status was used to classify patients into high‐risk/low‐risk disease, where high‐risk disease is defined as having at least two of the following: GS ≥8, at least three bone lesions, visceral metastasis.^8^

#### 2.1.2 Statistical analysis

Descriptive statistics were interpreted as means, medians, SDs, and proportions. Time to PSA progression and OS were assessed by the Kaplan‐Meier method. Survival comparisons were carried out by the Log‐rank test.

As the cutoff value for high vs low NLR is not well defined in literature, we opted to assess the correlation of NLR (< vs ≥ the median value) on the median time to PSA progression. In addition, we utilized cutoffs of 3 and 4 utilized and compared in the literature.^25^ We assessed the correlation of NLR at each of these cutoff points on time to PSA progression. In addition, we assessed the correlation of other‐disease related factors with time to PSA progression in univariate analysis, and then underwent a multivariate analysis for the significant factors (*P* < .05), utilizing the backward stepwise cox‐regression method.

We priori planned to utilize the cutoff value of NLR significantly associated with time to PSA progression in OS analysis. Univariate analysis to assess the possible impact of factors other than the NLR on OS, including: GS, age, serum PSA, visceral vs non‐visceral metastasis, ECOG PS, serum alkaline phosphatase, serum calcium, serum Hb, volume status, and risk status was conducted. All factors with *P* < .05 in the univariate analysis were forced into a multivariate analysis for OS utilizing the backward stepwise cox‐regression method. All *P*‐values <.05 were considered statistically significant. All statistical analyses were performed using SPSS version 21 (SPSS Inc, Chicago, Illinois).

## 3 RESULTS

### 3.1 Eligible patients

We identified a total of 280 patients with metastatic prostate cancer. Forty‐three (15%) were excluded because they presented as new cases of mCRPC. Seventeen (6%) were excluded because they came for a second opinion and continued treatment at other centers or lost follow up, 9 (3%) because the pathology did not confirm prostatic adenocarcinoma, 4 (1%) because of diagnosis of a concurrent second malignancy, and 4 (1%) because the metastasis was not detected on conventional imaging studies. In addition, 14 (5%) were excluded for missing data on complete blood counts prior to starting ADT. Thus, a total of 189 patients were included in the final analysis. Patients' characteristics are summarized in Table 1. The median age at presentation was 69 years (range: 40‐89), median serum PSA: 155 ng/mL (range: 1.1‐30 000), and median GS: 9 (range: 6‐10).

**TABLE 1:** Disease characteristics at initial presentation of included patients

Patients characteristics	N (%) or median (range)
De novo metastatic	181 (96%)
Prior local therapy	8 (4%)
Median age	69 (40–89)
Median baseline serum PSA	155 ng/mL (1.1–30 000)
Median GS	9 (6‐10)
Bone metastasis	174 (92%)
No bone metastasis	15 (8%)
One organ metastasis	104 (55%)
≥2 organ metastases	85 (45%)
Visceral metastasis	41 (22%)
Non‐visceral metastasis	148 (78%)
ECOG PS 0	82 (43%)
ECOG PS ≥1	83 (44%)
Missing	24 (13%)
Hb < 12 g/dL	50 (26%)
Hb ≥12 g/dL	139 (74%)
NLR < 4	150 (79%)
NLR ≥4	39 (21%)
High‐volume disease^a^	146 (77%)
Low‐volume disease	41 (22%)
Not assessable	2 (1%)
High‐risk disease^b^	144 (76%)
Low‐risk disease	44 (23%)
Not assessable	1 (<1%)

Abbreviations: ECOG PS, Eastern Cooperative Oncology Group Performance Status; GS, Gleason Score; NLR, neutrophil‐to‐lymphocyte ratio; PSA, prostatic‐specific antigen.

^a^ CHAARTED trial definition.

^b^ LATITUDE trial definition.

More than 90% (174/189) of patients had bone metastasis. Of 174 patients with bone metastasis, 37 (21%) had fewer than three bone lesions, and 135 (78%) had ≥3 bone lesions, whereas the number of lesions was not documented in 2 (1%). Approximately three‐quarters of patients had CHAARTED‐trial defined, and LATITUDE‐trial defined high‐volume and high‐risk status. Initial therapy administered was ADT alone for 134 patients (72%), ADT plus docetaxel (preplanned 6 cycles; CHAARTED protocol) for 48 (24%), and ADT plus abiraterone and prednisone for 7 (4%).

The median NLR was 2.7, mean NLR 3.2 (sd: 2.37; range: 0.5‐22). A total of 39 patients (21%) had NLR ≥4.

#### 3.1.1 NLR and time to PSA progression

**FIGURE 1:**
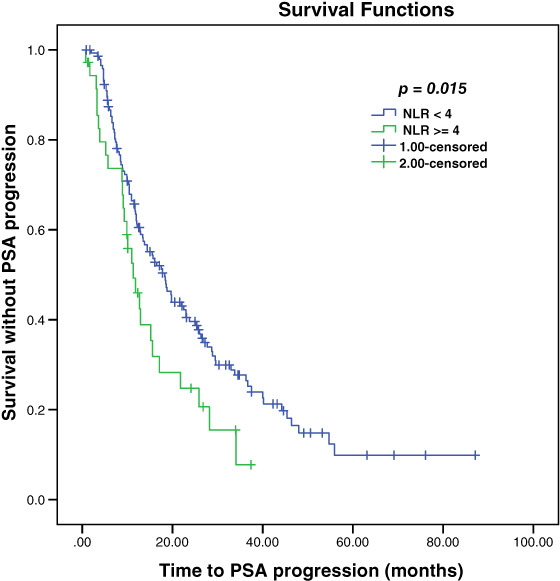
Kaplan‐Meier estimation of time to PSA progression according to NLR

The median time to PSA progression for the entire cohort was 15.6 months. Patients with NLR ≥4 had a shorter time to PSA progression compared to patients with NLR < 4; 11.3 and.18.3 months, respectively (*P* = .015) (Figure 1). We did not find any statistically significant difference of NLR on the median time to PSA progression using cutoff value of 3 (15.2 months for NLR ≥3 and 16.1 months for NLR < 3, *P* = .55). There was no impact of NLR ≥ median vs < median on time to PSA progression (15.5 and 16.1 months, respectively, *P* = .86).

On univariate analysis, ECOG PS≥1, serum ALP > ULN, Hb < 12 g/dL, high volume disease, and high‐risk status were also identified as significant factors predicting time to PSA progression (Table 2). We did not find any association of age, GS, the number of organ metastasis, visceral metastasis, serum calcium, and upfront docetaxel or abiraterone with time to PSA progression. In multivariate analysis, NLR ≥4 did not significantly predict time to PSA progression. However, ECOG PS ≥1, serum ALP > upper limit of normal, and Hb < 12 g/dL were identified as independent factors for shorter time to PSA progression (Table 3).

**TABLE 2:** Factors significantly predicting time to PSA progression in the univariate analysis

Factor	Median time to PSA progression (months)	*P*‐value
ECOG PS 0	28.2	<.001
ECOG PS ≥1	9.4	
ALP > ULN	9.4	<.001
ALP ≤ ULN	20.2	
Hb <12 g/dL	9.3	<.001
Hb ≥12 g/dL	18.4	
NLR ≥4	11.3	.015
NLR <4	18.3	
High volume disease^a^	12.1	.002
Low volume disease	26.3	
High risk disease^b^	12.0	.001
Low risk disease	28.2	

Abbreviations: ALP, Alkaline Phosphatase; ECOG PS, Eastern Cooperative Oncology Group Performance status; Hb, Hemoglobin; NLR, neutrophil‐to‐lymphocyte ratio; ULN, Upper Limit of Normal.

^a^ CHAARTED trial definition.

^b^ LATITUDE trial definition.

**TABLE 3:** Multivariate analysis for factors affecting time to PSA progression

Factor	HR (95% CI)	*P*‐value
ECOG PS ≥1	3.33 (2.18‐5.08)	<.001
ALP ≥ ULN	1.90 (1.30‐2.85)	.001
Hb < 12 g/dL	1.67 (1.10‐2.53)	.019
High volume disease^a^	1.14 (0.60‐2.19)	.67
High risk disease^b^	1.31 (0.69‐2.48)	.15
NLR ≥4	1.056 (0.65‐1.73)	.83

Abbreviations: ALP, Alkaline Phosphatase; ECOG PS, Eastern Cooperative Oncology Group Performance Status; Hb, hemoglobin; ULN, upper limit of normal.

^a^ CHAARTED trial definition.

^b^ LATITUDE trial definition.

#### 3.1.2 NLR and OS

**FIGURE 2:**
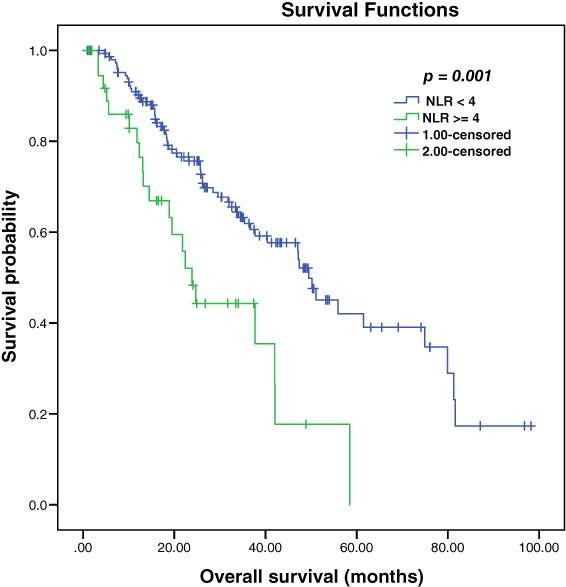
Kaplan‐Meier overall survival estimation according to NLR

At a median follow‐up of 26 months, the median OS for the entire cohort was 47.1 months. Median OS was inferior in patients with NLR ≥4 compared to patients with NLR < 4 [23.9 vs 49.5 months, *P* = .001] (Figure 2). We did not identify a significant difference of NLR on OS by median NLR or at cutoff 3 (*P*‐values were not significant).

Other factors that predicted OS in the univariate analysis included ECOG PS, volume status, risk status, serum ALP level, Hemoglobin level, and initial therapy administered (Table 4). Age, GS, serum calcium, visceral metastasis, prior local therapy, and the number of organ metastasis were not significant predictors of OS (*P*‐values were not statistically significant).

**TABLE 4:** Factors significantly predicting overall survival in the univariate analysis

Factor	Median OS (months)	*P*‐value
NLR ≥4	23.9	0.001
NLR <4	49.5	
ECOG PS ≥1	35.4	<.001
ECOG PS = 0	81.3	
High volume disease^a^	36.7	<.001
Low volume disease	61.5	
High risk disease^b^	37.7	.002
Low risk disease	81.6	
ALP > ULN	26.2	<.001
ALP ≤ ULN	58.5	
Hb <12 g/dL	20.5	<.001
Hb ≥12 g/dL	55.9	
ADT alone	40.4	.040
ADT plus docetaxel or abiraterone	Unreached	

Abbreviations: ADT, androgen deprivation therapy; ALP, alkaline phosphatase; ECOG PS, Eastern Cooperative Oncology Group Performance status; Hb, hemoglobin; NLR, neutrophil‐to‐lymphocyte ratio; OS, overall survival; ULN, upper limit of normal.

^a^ CHAARTED trial definition.

^b^ LATITUDE trial definition.

NLR≥4 remains an independent factor for inferior OS on multivariate analysis and was associated with three‐fold increased risk of death (Table 5).

**TABLE 5:** Multivariate analysis for factors affecting overall survival

Factor	HR (95% CI)	*P*‐value
ECOG PS ≥1	9.90 (4.0‐24.5)	<.001
ALP > ULN	1.39 (0.61‐3.17)	.43
Hb < 12 g/dL	5.75 (2.62‐14.29)	<.001
NLR ≥4	2.75 (1.01‐7.87)	.047
High‐volume disease^a^	4.83 (1.53‐15.27)	.005
High‐risk disease^b^	3.04 (0.98‐9.43)	.065
ADT alone	1.55 (0.50‐4.76)	.43

Abbreviations: ADT, androgen deprivation therapy; ALP, Alkaline Phosphatase; ECOG PS, Eastern Cooperative Oncology Group Performance Status; Hb, hemoglobin; ULN, upper limit of normal.

^a^ CHAARTED trial definition.

^b^ LATITUDE trial definition.

## 4 DISCUSSION

The importance of identifying pre‐treatment factors in patients with mCSPC that correlate with poor oncological outcomes stems from the fact that these patients might benefit from early treatment intensification. In this study, we assessed the impact of pre‐treatment NLR on oncological outcomes of patients with mCSPC and found that an elevated ratio was associated with inferior OS. Notably, after controlling for other patients and disease factors, elevated NLR remains an independent factor predicting inferior OS.

The role of several inflammatory markers such as platelet‐to‐lymphocyte ratio (PLR), CRP, albumin, and NLR have been extensively studied in the context of various malignancies, as these markers might reflect the interaction between the immune system and tumor microenvironment.^16^, ^17^, ^18^, ^26^, ^27^ For instance, the neutrophil count plays a major role in the tumor‐promoting activity, including cancer cell growth, and metastasis. While lymphocytes are suppressors of cancer progression.^26^, ^27^ Sejima et al emphasized the concept of “Fas ligand tumor counter‐attack,” where FasL in tumor cells induce apoptosis of cytotoxic T lymphocytes in the tumor microenvironment, thus higher NLR. Indeed, FasL is overexpressed in high‐grade prostate cancer and is associated with worse OS.^28^

Previous reports have assessed the prognostic value of NLR in the castration‐resistant stage of metastatic prostate cancer or those with non‐metastatic disease. In the meta‐analysis conducted by Yin et al, elevated NLR predicted poor OS (HR  =  1.57; 95% CI 1.41‐1.74; *P*   <  .001) and progression‐free survival (HR  =  1.97; 95% CI 1.28‐3.04; *p*   =  .002) of patients with mCRPC. On the contrary, NLR was not associated with oncological outcomes in patients with localized disease.^19^ Many other studies assessed the impact of NLR on treatment response to various treatment lines in mCRPC. For instance, elevated NLR (>2.14) was found to be associated with inferior OS in patients treated with enzalutamide.^29^ Other data showed a correlation between elevated NLR (> median value of 3.5) and inferior OS in patients treated with docetaxel in the first‐line setting.^20^ Mehra et al found a correlation between baseline NLR and clinical outcomes of patients with mCRPC treated with corticosteroids. In their study, a lower NLR was associated with superior PSA responses, and a higher NLR (log10) was associated with a shorter time to PSA progression (hazard ratio [HR], 9.5; 95% CI, 2.3‐39.9; *P* = .002).^30^ Moreover, baseline NLR ≥3.5 was predictive of lower PSA and radiologic responses to second‐line chemotherapy in mCRPC regardless of the use of corticosteroids.^31^ Most recently, a subanalysis of the CARD trial in mCRPC identified high baseline NLR (> median value of 3.38) as a predictor of a better response to cabazitaxel compared to Abiraterone/Enzulatamide.^32^

Identifying NLR as a prognostic factor in mCRPC has led to incorporating it in prognostic models and nomograms alongside other prognostic factors.^16^, ^23^ Such prognostic models may provide better insights on outcomes compared to the utilization of individual prognostic factors. Nevertheless, there is no data in the literature on the association of NLR with outcomes of patients with castration‐sensitive disease, and whether it could be useful to include the NLR in prognostic models. Therefore, we studied patients with castration‐sensitive metastatic disease and assessed the baseline NLR before the commencement of ADT. Importantly, we found a significant correlation of high NLR with OS. If these findings are externally validated in other cohorts, then the construction of prognostic models that include the NLR might give better insights on the outcomes of patients with mCSPC. In addition, such prognostic information may help investigate personalized risk‐adapted approaches of initial therapy selection.

Although our study is the first to identify a prognostic role of NLR in patients with mCSPC, there are many limitations that we acknowledge. First, the small sample size and the retrospective design of the study are recognized limitations. Second, the study includes patients treated over 12 years, in which disease presentation and management have evolved. Third, to date, there is still no consensus on the optimal NLR cutoff value. Nevertheless, the cutoff of 4 was selected as a potential significant cutoff according to our statistical plan and was not assessed as an exploratory analysis. This cutoff value selection was based on sensitivity analysis for selecting a cutoff of 3 vs 4 in a prospective cohort study in patients with solid metastatic tumors.^25^ According to the sensitivity analysis, HR value for OS was non‐significant at a cut‐off of 3.0 (HR: 1.34, 95% CI: 0.99‐1.32), but significant when the cutoff was 4.0 (HR: 1.53, 95% CI: 1.11‐2.10). Interestingly, we selected both cutoffs in our preplanned analysis, and our data were supportive of the sensitivity analysis data.

In conclusion, our data identified NLR ≥4 as an independent factor of inferior OS. However, NLR was not predictive of time to PSA progression. As such, if these findings are validated in other cohorts, elevated NLR may be used as a biomarker to predict OS in newly patients with mCSPC.

## CONFLICT OF INTEREST

The authors declare no potential conflict of interest.

## AUTHOR CONTRIBUTIONS

*Conceptualization; data curation; formal analysis; methodology; supervision; validation; writing‐original draft; writing‐review, and editing*, S.S.; *Data curation; formal analysis; validation; writing‐review and editing*, R. A.‐H.; *Conceptualization; data curation; formal analysis; writing‐review and editing*, F.A.; *Data curation; formal analysis; writing‐review and editing*, F.T. and A.A.‐T.; *Conceptualization; formal analysis; methodology; writing‐original draft; writing‐review and editing*, M.S.

## ETHICAL STATEMENT

This retrospective study was approved by the Institutional Review Board at King Hussein Cancer Center, Amman‐Jordan (approval of proposal no. 20 KHCC 96).

## Data Availability

The data used during the current study are available from the corresponding author on reasonable request.
